# Influence of Neonatal Hypothyroidism on Hepatic Gene Expression and Lipid Metabolism in Adulthood

**DOI:** 10.1371/journal.pone.0037386

**Published:** 2012-05-16

**Authors:** Ruymán Santana-Farré, Mercedes Mirecki-Garrido, Carlos Bocos, Luis A. Henríquez-Hernández, Nusrat Kahlon, Emilio Herrera, Gunnar Norstedt, Paolo Parini, Amilcar Flores-Morales, Leandro Fernández-Pérez

**Affiliations:** 1 Department of Clinical Sciences, Molecular and Translational Endocrinology Group, University of Las Palmas de GC – Cancer Research Institute of The Canary Islands (ICIC), Las Palmas de Gran Canaria, Spain; 2 Department of Biology, Faculties of Pharmacy and Medicine, University San Pablo-CEU, Madrid, Spain; 3 Department of Molecular Medicine and Surgery, Karolinska Institute, Stockholm, Sweden; 4 Division of Clinical Chemistry, Department of Laboratory Medicine and Molecular Nutrition Unit, Center for Nutrition and Toxicology, Karolinska Institute, Stockholm, Sweden; 5 Novo Nordisk Center for Protein Research, University of Copenhagen, Copenhagen, Denmark; Institut de Génomique Fonctionnelle de Lyon, France

## Abstract

Thyroid hormones are required for normal growth and development in mammals. Congenital-neonatal hypothyroidism (CH) has a profound impact on physiology, but its specific influence in liver is less understood. Here, we studied how CH influences the liver gene expression program in adulthood. Pregnant rats were given the antithyroid drug methimazole (MMI) from GD12 until PND30 to induce CH in male offspring. Growth defects due to CH were evident as reductions in body weight and tail length from the second week of life. Once the MMI treatment was discontinued, the feed efficiency increased in CH, and this was accompanied by significant catch-up growth. On PND80, significant reductions in body mass, tail length, and circulating IGF-I levels remained in CH rats. Conversely, the mRNA levels of known GH target genes were significantly upregulated. The serum levels of thyroid hormones, cholesterol, and triglycerides showed no significant differences. In contrast, CH rats showed significant changes in the expression of hepatic genes involved in lipid metabolism, including an increased transcription of PPARα and a reduced expression of genes involved in fatty acid and cholesterol uptake, cellular sterol efflux, triglyceride assembly, bile acid synthesis, and lipogenesis. These changes were associated with a decrease of intrahepatic lipids. Finally, CH rats responded to the onset of hypothyroidism in adulthood with a reduction of serum fatty acids and hepatic cholesteryl esters and to T3 replacement with an enhanced activation of malic enzyme. In summary, we provide *in vivo* evidence that neonatal hypothyroidism influences the hepatic transcriptional program and tissue sensitivity to hormone treatment in adulthood. This highlights the critical role that a euthyroid state during development plays on normal liver physiology in adulthood.

## Introduction

The thyroid hormones (THs) are essential for development, growth, and metabolism [1], [2], [3]. The physiological importance of the THs becomes evident under the condition of congenital-neonatal hypothyroidism (CH) [2]. If not treated immediately, CH has a profound impact on physiology and can permanently imprint neurological and endocrine systems, which, in turn, leads to mental retardation, growth arrest, and metabolic disturbances. During ontogeny, there exists a critical period when normal TH levels are required, and TH replacement after that period cannot correct the changes in gene expression that was caused by CH. This is exemplified by abnormal insulin-like growth factor binding protein (IGBFP)-2 expression in neonatal hypothyroid rats that can be corrected by TH replacement if started during the first week of life but not later [4].

Most of the effects of the THs result from their interaction with TH receptors (TR), which can bind to the TH response element (TRE) located in several target genes [1]. A TRE is present upstream of the rat GH gene [5], the product of which has a significant role in body growth and metabolism in postnatal life [3]. GH expression is induced by T3 and reduced in hypothyroid states [6], [7], [8]. In fact, the growth-promoting effects of T3 can be largely explained by its ability to induce normal GH secretion and to regulate the expression patterns of the GH receptor in the liver and the IGF-I receptor in peripheral tissues [9]. Therefore, the THs might exert physiological actions on the liver through a direct transcriptional regulation of several target genes [1] and indirect mechanisms (e.g., modulating actions of GH and metabolic sensors such as LXR) [6], [7], [8], [9], [10], [11]. In addition, it has been shown that the effects of the THs on glucose metabolism might also be dependent on their actions in the brain, which affect the autonomic control of peripheral metabolic tissues such as the adipose tissue in addition to the liver [12], [13], [14]. These findings suggest that CH could cause permanent alterations in peripheral metabolic tissues through irreversible developmental alterations in the brain. The THs clearly have some lipid-lowering actions, as demonstrated by recent human studies [15]. For example, the ability of THs to reduce plasma LDL-c levels has been explained by its capacity to increase the expression of LDL receptors and the activities of lipid-lowering enzymes in the liver. THs increase the number of LDL receptors without necessarily modifying their transcription, which supports a post-transcriptional regulation of these proteins [16]. Some lipid-lowering enzymes are direct transcriptional targets. These are exemplified by the CYP7A1 gene [17], [18], the product of which has a major cholesterol-lowering activity. Malic enzyme (ME) and fatty acid synthase (FAS), two critical regulators of lipogenesis, are also directly regulated by the THs [19], [20], [21]. In contrast, the effects of THs on other lipogenic genes such as sterol regulatory element-binding protein (SREBP)-1c are more controversial: THs induced SREBP1c expression in the livers of rats [22], [23], whereas they strongly downregulated SREBP1c in the livers of mice [24].

In rats, the serum concentrations of the THs are low at birth and increase progressively, reaching adult levels at approximately the third week of life when the maturation of the hypothalamus-pituitary-thyroid axis is achieved [25]. Administration of the antithyroid drug methimazole (MMI) to pregnant rats before the onset of fetal thyroid function is known to abolish both the maternal and fetal thyroid function [26]. In this context, exposure to MMI during the fetal-neonatal period of life is an attractive model to study the influence of CH on the adult pattern of genes that are under the control of the THs during rat development [4]. In addition, the disturbances in endocrine status during the neonatal period of life may affect the susceptibility to chronic diseases or biological insults in adulthood [14], [27], [28], [29], [30]. However, the precise mechanism whereby CH influences liver physiology in adulthood remains poorly understood. We hypothesized that CH might lead to changes in TH and/or GH action during development that have physiological repercussions in adulthood. Therefore, there were two aims of this study. First, we assessed the influence of CH on the somatotropic axis, liver gene expression, and serum and hepatic lipid biomarkers in adulthood. Second, we investigated how rats, which were transiently exposed to CH, adapted to the biological insult of the onset of hypothyroidism in adulthood. In the present study, we provide *in vivo* evidence that exposure to CH alters postnatal development, which influences the liver transcriptional program that is associated with altered lipid homeostasis and tissue sensitivity to hormones.

## Materials and Methods

### Materials

Recombinant human GH was kindly donated by Pfizer Laboratories (Spain). Tri-Reagent and the rest of the products cited in this work were purchased from the Sigma Chemical Co. (St. Louis, MO), unless otherwise indicated.

### Animal study design

This study was carried out in strict accordance with the recommendations in the Guide for the Care and Use of Laboratory Animals of the University of Las Palmas de G.C. and conducted in accordance with European and Spanish laws and regulations. The protocol was approved by the Committee on the Ethics of Animal Experiments of the University of Las Palmas de G.C. (permit number: 2006-07824). All efforts were made to minimize suffering. Male Sprague-Dawley rats (n = 6 per group) were used throughout these experiments. Animals were kept under a constant dark/light cycle in a controlled temperature (21–23°C) environment and had free access to a standard diet (A04 SAFE; Panlab, Barcelona, Spain) and tap water throughout the experiment. For mating purposes, four females were housed overnight with two males starting at 21:00 h. Females were checked by 7:00 h the next morning, and the presence of a vaginal plug was designated as gestational day (GD) 0. Within 24 h after birth, excess pups were removed so that 8 pups were kept per dam. The indirect MMI exposure method (i.e., through maternal milk) utilized in this experiment has been extensively employed to induce a transitory congenital-neonatal hypothyroidism (CH) for the determination of short- and long-term physiological effects [4], [26], [29], [30]. Briefly, 0.02% MMI was administered in the drinking water for pregnant rats from GD12 until weaning at post-natal day (PND) 30, which is when pups were mature enough to support their own nutritional needs. The MMI-containing water was changed twice per week. Untreated rats served as concurrent euthyroid and age-matched controls (INTACT). Rats that were weight paired with the CH groups were also included to control for the effects of reduced body weight on hepatic gene expression as follows: 1) WP30, weight-paired rats with CH group on PND30, corresponds to animals that were sacrificed on PND24 and 2) WP, weight-paired rats with CH group on PND80, which were sacrificed on PND53. For the generation of adult hypothyroid rats (TX), 0.05% MMI was added to the drinking water for 3 weeks [23] starting at PND60 until sacrifice at PND80. Thus, two additional groups were created as follows: 1) adult TX rats without CH (TX/−CH) and 2) adult TX rats with CH (TX/+CH). A schematic diagram of this experimental model is shown in Supplementary File S1. The presence of hypothyroidism was corroborated by monitoring the body weight and serum levels of T3 and T4. During the last week of life, TX rats were either injected with T3 (20 µg/kg b.w.) as a single daily ip injection or with GH (0.4 mg/kg/day) divided into two daily sb injections that were carried out at 08:00 h and 20:00 h. T3 was dissolved in a minimum volume of 0.01 N NaOH and was brought up to the appropriate concentration with sterile saline. In parallel, TX animals received equivalent amounts (0.20 ml) of the vehicle alone (VEH). Twenty-four hours (in the case of T3) or twelve hours (in the case of GH) after the last injection, animals were sacrificed by exsanguination after pentothal anesthesia. Twelve hours before the rats were sacrificed, the diet was removed from the cages to minimize the effect of food. Serum samples were collected and stored at −80°C until analysis. Portions of the liver were snap frozen in liquid nitrogen and stored at −80°C until being processed for mRNA or biochemical analysis.

### Growth and food intake analysis

Body weights, tail lengths, and food intake were measured once a week for all animals. The measurement of tail length was used for monitoring growth [31], [32], [33], [34] . The percentages of weight rate (WR) or tail rate (TR) were calculated by the following formulas: WR = [(W (g) (new) – W (g) (old)/W (g) (old)]*100 or TR = [(T (cm) (new) – T (cm) (old)/T (cm) (old)]*100, respectively. Food consumption was estimated by subtracting the amount of food left on the grid from initial food weight. Food spilled on the floor of the cage was not weighed, but spillage was minimal because the diet was supplied as pellets. Mother and pups were housed in the same cage until weaning on PND30. Then, weaned pups were housed in pairs to control for food efficiency. The weekly caloric intake was calculated on the basis of food consumption x caloric value of the diet (2900 kcal/g). Feed efficiency (FE), which denotes the body weight increase per gram of food consumed or the ability to transform calories consumed into body weight [35], was calculated by the following formula: mean body weight gain (g)/total caloric intake. The weights of the liver, heart, and kidneys as well as the organ weight/total body weight ratios were also calculated on PND30 and PND80.

### Serum IGF-I, TSH, T3, and T4 analysis

The **s**erum levels of IGF-I (Quantikine; R&D Systems) and TSH (Gentaur Molecular Products, Belgium) were determined by using rat immunoassays and following the manufacturers' recommendations. All of the samples were assayed together, and each sample was assayed in duplicate. The serum free T4 and T3 concentrations (ng/dl) were measured in duplicate by an enzyme immunoassay (Access Immunoassay Systems, Beckman Coulter, Inc., USA) with detection limits of 0.60 ng/dl and 88 ng/dl, respectively.

### Serum lipid analysis

Lipoproteins were separated essentially as previously described [36] using a Superose®-12 PC 3.2/30 column (Pharmacia Biotech, Uppsala, Sweden). Serum from each animal (2.5 µL) was separated for cholesterol and triglycerides, which were subsequently assayed on-line. Total cholesterol and triglycerides were assayed using cholesterol and triglyceride colorimetric enzymatic kits (Roche/Hitachi Diagnostic GmbH, Mannheim, Germany). The serum triglyceride value for each animal was normalized to the respective glycerol content. Absorbance was continuously measured at 500 nm, and data were collected every 10 s using EZ Chrom™ software (Scientific Software, San Ramon, CA).

### Hepatic lipid analysis

Frozen liver aliquots were used for lipid extraction [37], and aliquots of lipid extracts were quantified after separation by one-dimensional TLC [38] and image analysis using the G5-700 Bioimage TLC scanner (Bio-Rad, CA). The spots were quantified as integrated optical density against an internal standard of cholesteryl formate and against calibration curves of the different lipid standards.

### RNA isolation, cDNA microarray, probe preparation, and hybridization

Total RNA was isolated by the homogenization of frozen liver as previously described [23]. All samples were treated with RNase-free DNase (Promega, Madison, WI). The RNA yields were measured by UV absorbance, and the quality of total RNA was analyzed with a 2100 Bioanalyzer (Agilent Technologies, Palo Alto, CA). A microarray containing 27,000 rat 70-mer oligo probe sets, which was produced at the KTH Microarray Center (www.biotech.kth.se), was used to evaluate the effects of CH on liver gene expression in adulthood. Five micrograms of high-quality total RNA from liver was reversed-transcribed, labeled, and hybridized according to the manufacturer's protocol (Pronto™ Plus System; Promega). After 16 h of hybridization, the slides were scanned using a GenePix Microarray Scanner (Axon Instruments, CA). Four independent hybridizations were performed to compare individual animals from the CH group (n = 4) with those from the INTACT group (n = 4) on PND80 for a total of four analyses.

### Data processing and analysis

Image analysis was performed using GenePix Pro 6.0 software (Axon Instruments, Union City, CA) as previously described [23]. The LOWESS (Locally Weighted Scatter Plot Smoother) method in the SMA (Statistics for Microarray) [39] package (www.bioconductor.org) was used to normalize the raw data. The probe sets not present in at least three of the four chips were considered as meaningless and therefore were eliminated to reduce data complexity. Identification of differentially expressed genes was performed using the SAM (Significance Analysis for Microarrays) statistical technique [40]. A *q* value was assigned to each of the detectable genes in the array. This value is similar to a *P*-value and measure the lowest false discovery rate (FDR) at which the differential expression of a gene is considered significant. A minimal FDR of 0.05 was assigned for each gene. An additional selection requirement was added to this statistically based criterion, which was based on the absolute changes in the gene expression ratios. A value of 1.5 (50%) (log_2_ ratio CH/INTACT≥|0.58|) was chosen to describe ratios as up- or downregulated. Functional classifications (Gene Ontology) and pathway analysis (KEGG) of differentially expressed genes that were affected by CH were performed by using the web-based tool DAVID [41]. All microarray data are MIAME compliant, and the raw data have been deposited in Gene Expression Omnibus database (www.ncbi.nlm.nih.gov/geo).

### Gene expression analysis by real-time quantitative PCR (qPCR)

The mRNA expression levels of genes were measured using qPCR as previously described [23]. Briefly, 2 µg of total RNA was treated with RNase-free DNase I (Promega) and reverse transcribed by using an iScriptTM kit (Bio-Rad Laboratories). Two microliters of cDNA served as a template in a 20-µl qPCR reaction mix containing the primers and SYBR Green PCR Master mix (Diagenode, Belgium). Quantification of the gene expression was performed with an ABI PRISM® 7000 SD PCR System. A dissociation protocol was performed to assess the specificity of the primers and the uniformity of the PCR generated products. Exon-specific primers were designed by the Primer 3 program [42] and are listed in Supplementary File S2. The level of individual mRNAs measured by qPCR was normalized to the level of the housekeeping genes cyclophilin and ribosomal 28S by using the Pfaffl method [43]. For graphing purposes, the relative expression levels were scaled such that the expression of the INTACT control group equalled one.

### Statistical analysis

The data are expressed as the means ± SD. The significance of differences between the groups was tested by either a two-tailed Student's *t* test or a one-way ANOVA, which was followed by post hoc comparisons of the group means according to the GraphPad Prism 5 program (GraphPad Software, San Diego, CA). A two-tailed Student's *t* test was performed on PND80 for body weight and tail length to assess the effect of CH and the completeness of recovery. Statistical significance was reported if *P*<0.05 was achieved.

## Results

### Neonatal hypothyroidism delays body growth development and is followed by catch-up growth

There were no significant MMI treatment-related differences in dam's body weights and intake of feed and drinking water throughout gestation (data not shown). There were no apparent MMI treatment-related effects on the offspring's body weights over the 3–4 PND. As expected [4], [26], the INTACT pups on the tap water recorded normal size and growth during development, while defects due to the MMI treatment in CH rats were evident by a reduction in body weight (Supplementary File S3; panel A) and tail length (Supplementary File S3; panel B) from the second week of life. Upon weaning on PND30, biochemical hypothyroidism was shown, and significantly low circulating T3 (pg/ml) (9.93±0.66 *vs.* 7.80±0.83; *P* = 0.0008) and elevated TSH (ng/ml) (2.73±2.04 *vs*. 6.72±2.5; *P* = 0.012) levels were found in CH rats in comparison with the age-matched INTACT group. In addition, circulating IGF-I (ng/ml) levels were reduced by 55% in the CH group (716.33±132.62 vs. 323.25±45.97; *P* = 0.0001). Furthermore, the mRNA levels of ME (0.15±0.10 vs. 1±0.04; *P* = 0.0001) and Spot14 (0.30±0.10 vs. 1±0.02; *P* = 0.0001), two genes positively regulated by TH [1], were also markedly reduced in the CH group. In liver, the fetal expression pattern of high IGFBP-2 levels was replaced by the adult pattern of low levels of IGFBP-2 only in the presence of normal levels of TH during rat development [4]. CH rats expressed higher levels of IGFBP2 mRNA in comparison with the INTACT or WP control rats (Supplementary File S3; panel C). Collectively, these data support the presence of neonatal hypothyroidism on PND30.

**Figure 1:**
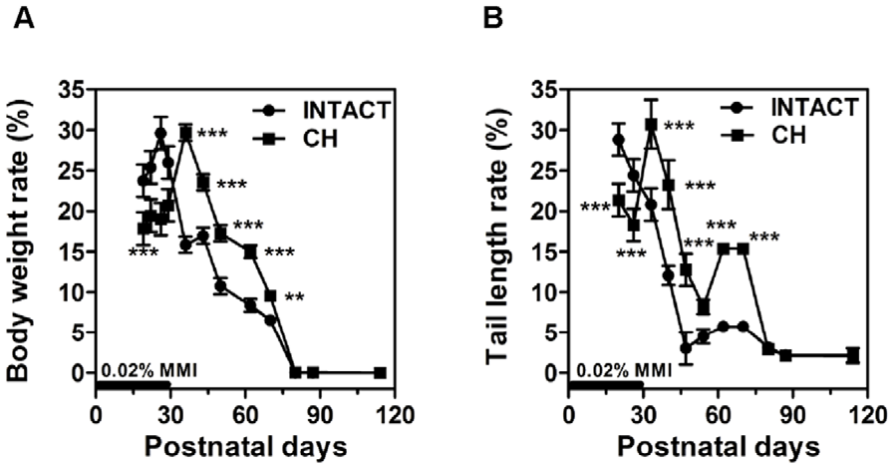
Effects of neonatal hypothyroidism on body growth development. Percentages of body weight rate (A) or tail length rate (B) were measured at 7-d intervals as described in Material and Methods. Results are expressed as mean ± SD from six individual animals in each group. **, *P*<0.01, ***, *P*<0.001 for comparison with INTACT group.

**Figure 2:**
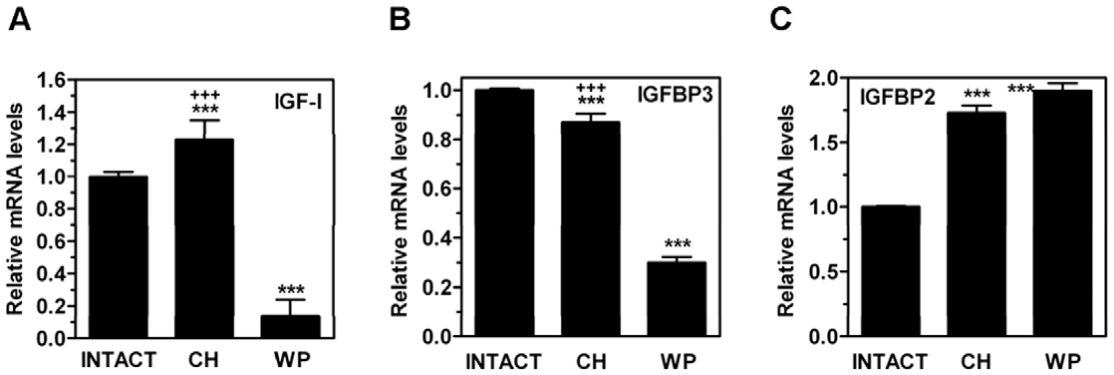
Effects of neonatal hypothyroidism on mRNA expression levels of IGF-I and IGFBP genes in adult rat liver. On PND80, the hepatic mRNA levels of IGF-I (A), IGFBP3 (B), and IGFBP2 (C) were measured by qPCR in rats exposed to neonatal hypothyroidism (CH), age-matched (INTACT) or weight-paired (WP) control groups. The mean mRNA expression level of each gene in the INTACT group is defined as 1, with all other expression values reported relative to this level. Bars represent mean ± SD from at least six individual animals. ***, *P*<0.001 for comparison with INTACT group. **+++**, *P*<0.001 for comparison with WP group.

**Figure 3:**
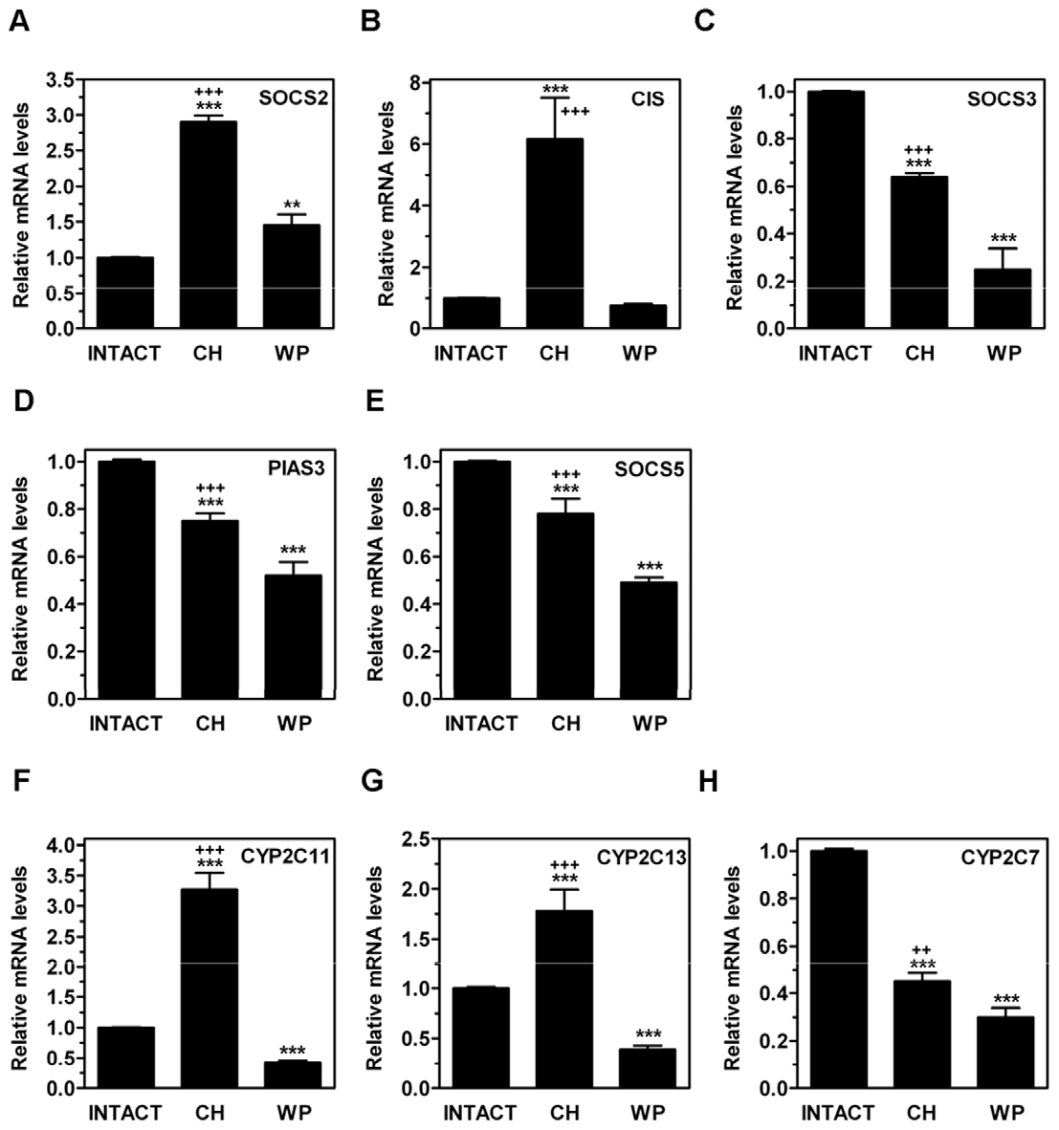
Effects of neonatal hypothyroidism on mRNA expression levels of SOCS/CIS and male predominant genes in adult rat liver. On PND80, the hepatic mRNA levels of SOCS2 (A), CIS (B), SOCS3 (C), PIAS3 (D), SOCS5 (E), CYP2C11 (F), CYP2C13 (G) and CYP2C7 (H) were measured by qPCR in rats exposed to neonatal hypothyroidism (CH), age-matched (INTACT) or weight-paired (WP) control groups. The mean mRNA expression level of each gene in the INTACT group is defined as 1, with all other expression values reported relative to this level. Bars represent mean ± SD from at least six individual animals. ***, *P*<0.001 for comparison with INTACT group. **++**, *P*<0.01; **+++**, *P*<0.001 for comparison with WP group.

Once the MMI treatment was discontinued on PND30, the animals gradually recovered and significant catch-up (defined as growth rate that is greater than normal for age after a period of growth inhibition) [44] occurred in terms of body weight (Supplementary File S3; panel D) and tail length (Supplementary File S3; panel E). It was evident in all hypothyroid rats that, **w**hen MMI was withdrawn, they rapidly began to increase their growth rates in a compensatory manner to make up for their deficits and to catch up with the normal age-matched control rats. These results suggest that, in agreement with what has been previously shown [45], [46], [47], [48], the plasma levels of THs were rapidly recovered (i.e., within 2–4 days after discontinuation of MMI treatment). Interestingly, the feed efficiency was significantly increased in CH rats (Supplementary File S3; panel F). On PND80, a statistically significant difference remained in body mass (g) (430±29.69 vs. 333±29.72; *P* = 0.002) (Supplementary File S3; panel A), tail length (cm) (21.9±0.62 vs. 20.3±0.73; *P* = 0.002) (Supplementary File S3; panel B), and circulating IGF-I levels (ng/ml) (1541.24±76.74 vs. 1305.15±126.27; *P* = 0.003) between INTACT and CH groups, respectively, indicating that growth was incomplete. Importantly, at the time of sacrifice, the growth rate, measured as a percentage of body weight gain (Fig. 1A) or tail length gain (Fig. 1B), was similar between the two groups, which suggests that the CH animals may have finished a period of rapid catch-up growth. The weights of the liver and kidneys at the end of the study (i.e., PND80), corrected by total body weight, were similar in the CH rats in comparison with age-matched INTACT rats (data not shown), which also suggests that the compensatory growth of organs in relation to body weight was achieved. Furthermore, on PND80, the serum levels of T3 (ng/dl) (45.15±5.77 vs. 47.52±3.17), T4 (ng/dl) (1.94±0.27 vs. 1.52±0.18), total cholesterol (mM) (1.54±0.15 vs. 1.65±0.21), and triglycerides (mM) (1.20±0.23 vs. 1.18±0.28) in the CH group did not differ from the INTACT animals. Subtle changes in hepatic IGF-I (Fig. 2A) and IGFBP-3 (Fig. 2B) mRNA levels together with increased levels of IGFBP-2 (Fig. 2C), a gene that is a marker of delayed development or hypothyroidism in rats [4], [49], were also shown on PND80. Because the expression of IGFBP-2 mRNA levels was similar in the CH and WP groups, this result indicates that the overexpression is a consequence of delayed growth and low body weight rather than a long-lasting consequence of CH. However, in comparison with age-matched INTACT rats, several genes remained unaltered or were upregulated in CH rats whereas they were significantly downregulated in the WP control group (Fig. 3–5). Collectively, these results suggest that the induction of neonatal hypothyroidism alters the normal development of the liver gene expression program, which may impact GH-related liver functions.

**Figure 4 pone-0037386-g004:**
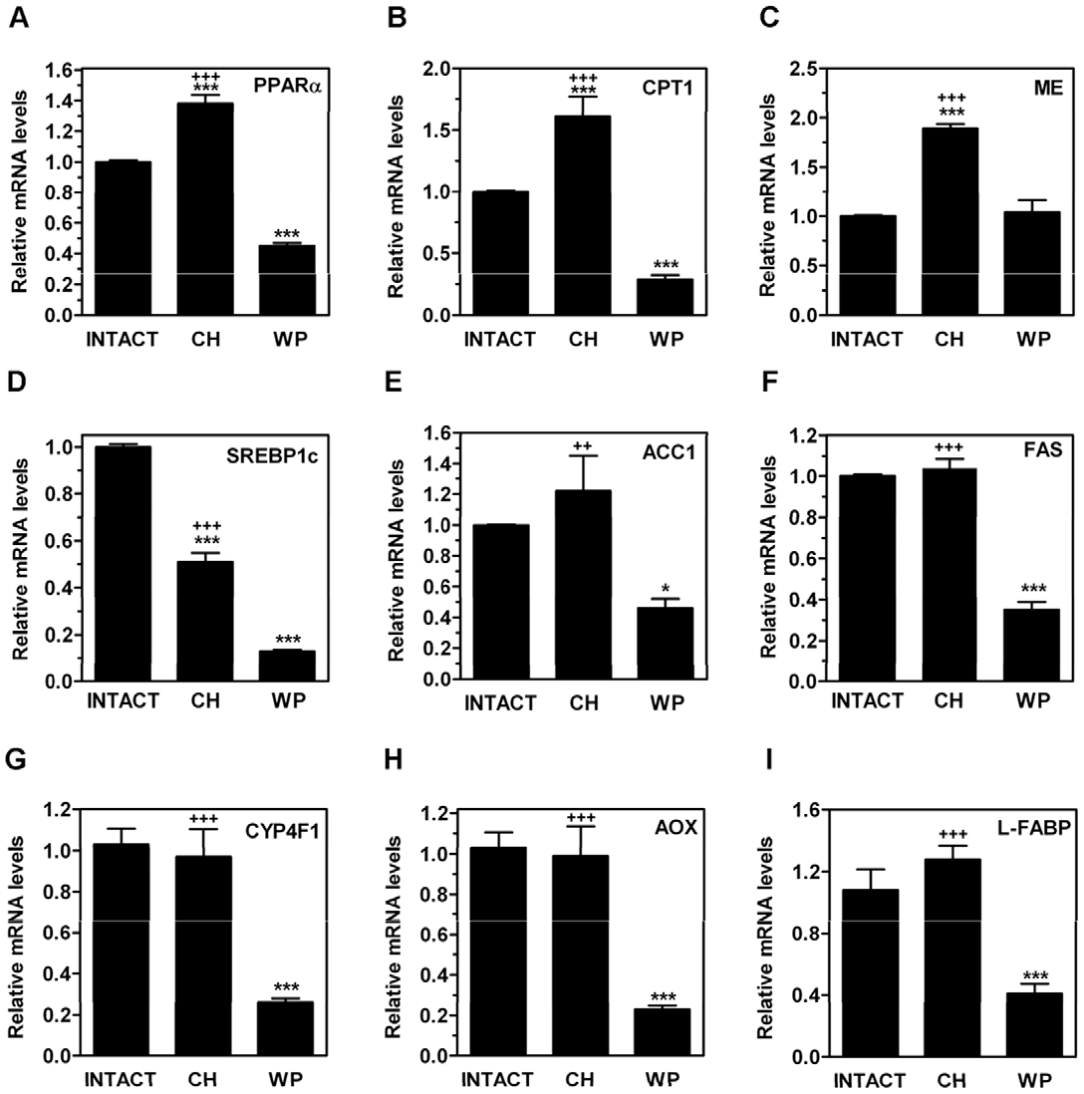
Effects of neonatal hypothyroidism on mRNA expression levels of genes related with lipid metabolism in adult rat liver. On PND80, the hepatic mRNA levels of PPARα (A), CPT1 (B), ME (C), SREBP1c (D), ACC1 (E), FAS (F), CYP4FI (G), AOX (H), and L-FABP (I) were measured by qPCR in CH, age-matched INTACT, or weight-paired (WP) control groups. The mean mRNA expression level of each gene in the INTACT group is defined as 1, with all other expression values reported relative to this level. Bars represent mean ± SD from at least five individual animals. *, *P*<0.05; ***, *P*<0.001 for comparison with INTACT group. **++**, *P*<0.01; **+++**, *P*<0.001 for comparison with WP group.

**Figure 5:**
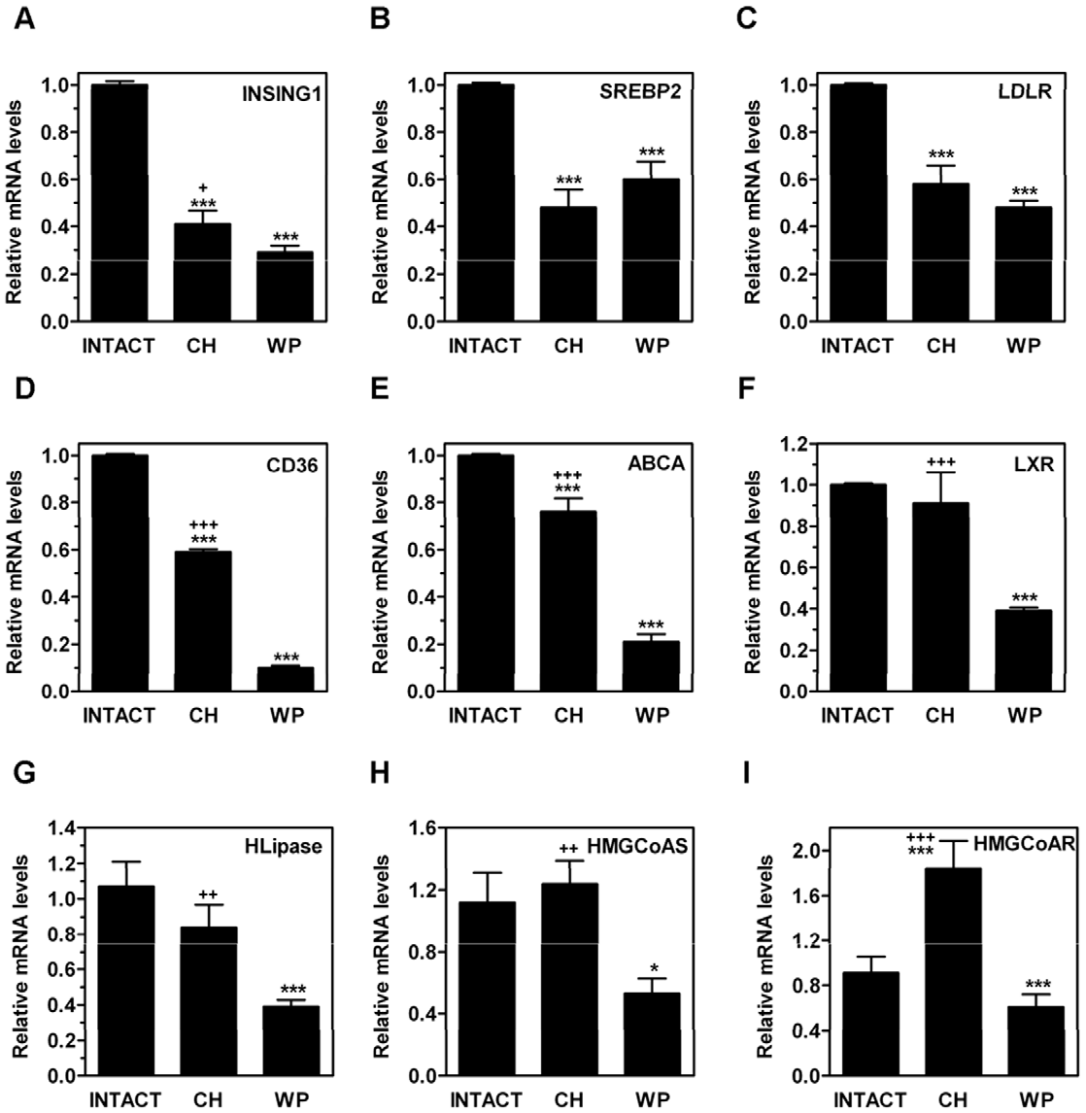
Effects of congenital hypothyroidism on mRNA expression levels of INSIG-1, SREBP2, LXR, and genes involved in lipid transport in adult rat liver. On PND80, the hepatic mRNA levels of INSIG1 (A), SREBP2 (B), LDLR (C), CD36 (D), ABCA (E), LXR (F), HLipase (G), HMGCoAS (H), and HMHCoAR (I) were measured by qPCR in CH, age-matched INTACT or weight-paired (WP) control groups. The mean mRNA expression level of each gene in the INTACT group is defined as 1, with all other expression values reported relative to this level. Bars represent mean ± SD from at least five individual animals. *, *P*<0.05; ***, *P*<0.001 for comparison with INTACT group. **+**, *P*<0.05; **++**, *P*<0.01; **+++**, *P*<0.001 for comparison with WP group.

### Influence of neonatal hypothyroidism on Growth Hormone-regulated genes

An altered growth rate or a long-lasting influence on somatotropic axis (i.e., GH, liver and IGF-I) could be related to the effects of CH on the hepatic GH signaling system [50], [51]. To test this hypothesis, we next analyzed the mRNA expression levels of the GH-regulated genes CIS and SOCS2, which act as negative regulators of GH signaling. On PND30, the CIS mRNA expression level was significantly upregulated (*P*<0.001) in CH rats (9.93±4.45) in comparison with the INTACT (1±0.03) and WP30 (0.87±0.67) groups, whereas SOCS2 remained unchanged. However, on PND80, SOCS2 (Fig. 3A) and CIS (Fig. 3B) were upregulated whereas SOCS3 (Fig. 3C) and additional GH-independent negative regulators of STAT-mediated signaling such as PIAS3 (Fig. 3D) and SOCS5 (Fig. 3E) were downregulated. We next explored whether the increased hepatic expression of SOCS2 and CIS mRNA levels in adult rats exposed to CH was associated with a negative regulation of GH target genes or instead reflected enhanced GH signaling. CYP2C11 (Fig. 3F) and CYP2C13 (Fig. 3G), two biomarkers of the male gene expression pattern in rats that are under GH control [52], were overexpressed in CH in comparison with the INTACT or WP groups, whereas the female-predominant CYP2C7 gene was downregulated (Fig. 3H). These results show that CH influences the mRNA regulation of GH target genes in the adult liver. The absence of major effects on IGF-I expression suggests the presence of complex transcriptional mechanisms *in vivo* that deserve further research.

### Neonatal hypothyroidism influences the liver gene expression program in adulthood, and it is associated with decreased levels of intrahepatic lipids

To better understand the influence of transient neonatal hypothyroidism on adult liver physiology, we next carried out an exploratory analysis of the global gene expression changes in the CH group on PND80 compared to INTACT rats by using DNA microarrays. We identified the set of genes that were differentially regulated in the CH group and analyzed the biological processes represented based on gene annotations in the Gene Ontology (www.ncbi.nlm.nih.gov/geo) and the KEGG pathway databases [41]. These exploratory analyses indicated that genes with altered expression in the CH group were functionally related to lipid metabolism (*P* = 0.0001). In particular, “PPARα” was the signaling pathway (KEGG) most represented among the CH regulated genes, suggesting alterations in hepatic lipid metabolism even in the absence of overt changes in circulating cholesterol and triglyceride levels (see below). Accordingly, our study showed that the hepatic content (mg/g of tissue; n = 6) of triglycerides (3.91±0.6 vs. 1.49±0.18; *P*<0.001), cholesteryl esters (0.30±0.02 vs. 0.21±0.04; *P*<0.001), free fatty acids (FFA) (0.18±0.03 vs. 0.13±0.004; *P*<0.05), and phospholipids (57.8±4.1 vs. 48.7±8.8; *P*<0.05) were significantly reduced in CH in comparison with INTACT rats. However, no changes in the free cholesterol (2.4±0.2 vs. 2.1±0.3) content (mg/g of tissue) were observed.

In order to determine whether the decreased hepatic content of lipids in CH rats was due to increased lipid catabolism, decreased lipid synthesis, increased lipid excretion, or some combination of these mechanisms, we analyzed the hepatic mRNA levels for proteins mediating these processes by using qPCR. First, Figure 4A shows that the mRNA levels of PPARα, a master regulator of β-oxidation, were upregulated in the CH group. Similar results were observed when measuring the mRNA expression of carnitine palmitoyl transferase-1 (CPT1) (Fig. 4B), a PPARα target gene coding for a protein that is important for transferring fatty acyl-CoAs into the mitochondria for β-oxidation [53]. In contrast, PPARα (Fig. 4A) and CPT1 (Fig. 4B) were downregulated in the WP group. Phosphoenolpyruvate carboxykinase (PEPCK), a rate-limiting gene in gluconeogenesis, remained unaltered in the CH group, whereas it was significantly reduced in the WP control group (data not shown).

**Figure 6:**
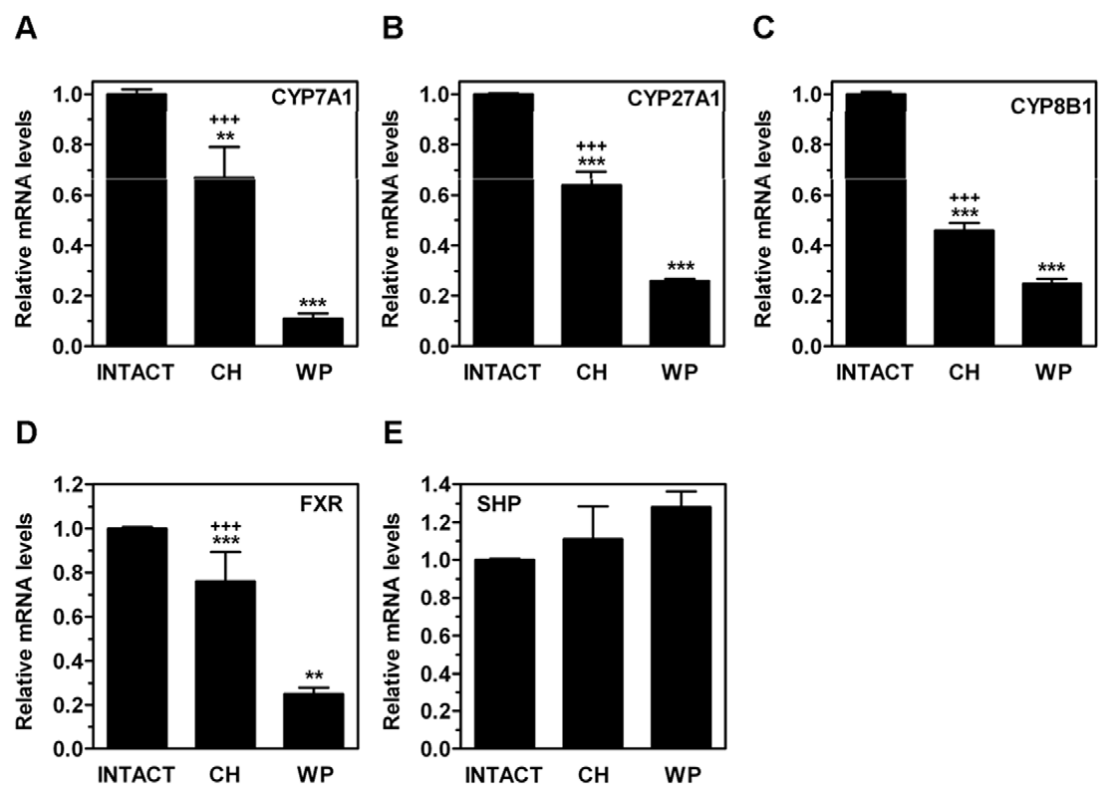
Effects of congenital hypothyroidism on mRNA expression levels of genes involved in bile acid synthesis in adult rat liver. On PND80, the hepatic mRNA levels of CYP7A1 (A), CYP27A1 (B), CYP8B1 (C), FXR (D), and SHP (E) were measured by qPCR in CH, age-matched INTACT or weight-paired (WP) control groups. The mean mRNA expression level of each gene in the INTACT group is defined as 1, with all other expression values reported relative to this level. Bars represent mean ± SD from at least five individual animals. **, *P*<0.01; ***, *P*<0.001 for comparison with INTACT group. **+++**, *P*<0.001 for comparison with WP group.

ME is a TH-regulated enzyme that converts malate into pyruvate to generate NADPH which is destined for lipogenesis and other biosynthetic processes [54]. We found that the ME mRNA levels (Fig. 4C) were upregulated in the CH rats on PND80, but the levels of SREBP1c (Fig. 4D), a master regulator of lipogenesis, were significantly downregulated. The SREBP1c target genes, acetyl-CoA carboxylase-1 (ACC1) (Fig. 4E), the product of which is the rate-limiting metabolite for fatty acid biosynthesis and inhibits CPT-1 [55], and fatty acid synthase (FAS) (Fig. 4F), as well as several genes known to be regulated by PPARα such as CYP4F1 (Fig. 4G), palmitoyl-CoA oxidase (AOX) (Fig. 4H) and liver fatty acid-binding protein (L-FABP) (Fig. 4I), were not affected in the CH group whereas they were significantly reduced in the WP control group. The steady-state mRNA level for insulin-induced gene 1 (INSIG-1) (Fig. 5A), an SREBP target, was also reduced in the CH rats. Overall, our data do not support the activation of a lipogenic program in the CH rats. Interestingly, the hepatic mRNA expression levels of SREBP2 (Fig. 5B) and its target gene LDLR (Fig. 5C), the product of which is involved in hepatic VLDL uptake, were 2–3-fold lower in the CH rats compared with the INTACT rats. Similar results were obtained for the gene expression levels of CD36 (Fig. 5D), which is involved in the uptake of FFA and microsomal triglyceride transfer protein (MTTP) (data not shown), which is involved in triglycerides assembly, indicating that diminished uptake of fatty acids may contribute to the reduced hepatic TG levels observed in the CH rats [56]. ABCA1 (Fig. 5E), a transporter responsible for cholesterol efflux, was also reduced whereas LXR (Fig. 5F), hepatic lipase (HL) (Fig. 5G), and HMGCoA-S (Fig. 5H) remained unchanged. HMGCoA-R (Fig. 5I) was upregulated in CH group. In contrast, the mRNA expression levels of LXR, HL, HMGCoA-R, and HMGCoA-S were significantly downregulated in the WP group. In addition, the mRNA expression levels of enzymes responsible for the catabolism of cholesterol such as 7α-hydroxylase (CYP7A1) (Fig. 6A), which is the rate-limiting enzyme in hepatic bile acid synthesis, sterol 27 hydroxylase (CYP27A1) (Fig. 6B), and 12α-hydroxylase (CYP8B1) (Fig. 6C), which is an enzyme involved in cholic acid synthesis [57], [58], were downregulated in the CH and in the WP groups in comparison with the INTACT rats. Farnesoid X receptor (FXR) (Fig. 6D), a receptor for bile acids [57], was also downregulated in the CH rats. In contrast, small heterodimer partner (SHP) (Fig. 6E), a gene induced by FXR, remained unchanged, which suggests that there is low activity of the bile acid-FXR signaling pathway. Collectively, these data indicate that a transient neonatal exposure to hypothyroidism causes changes in lipid metabolism in the liver and, particularly, in the cholesterol metabolism pathway that persist long after removal of the original insult, in animals that are euthyroid and exhibit similar growth rate as intact rats.

### Neonatal hypothyroidism influences the hepatic response to hormone replacement in adulthood

**Figure 7:**
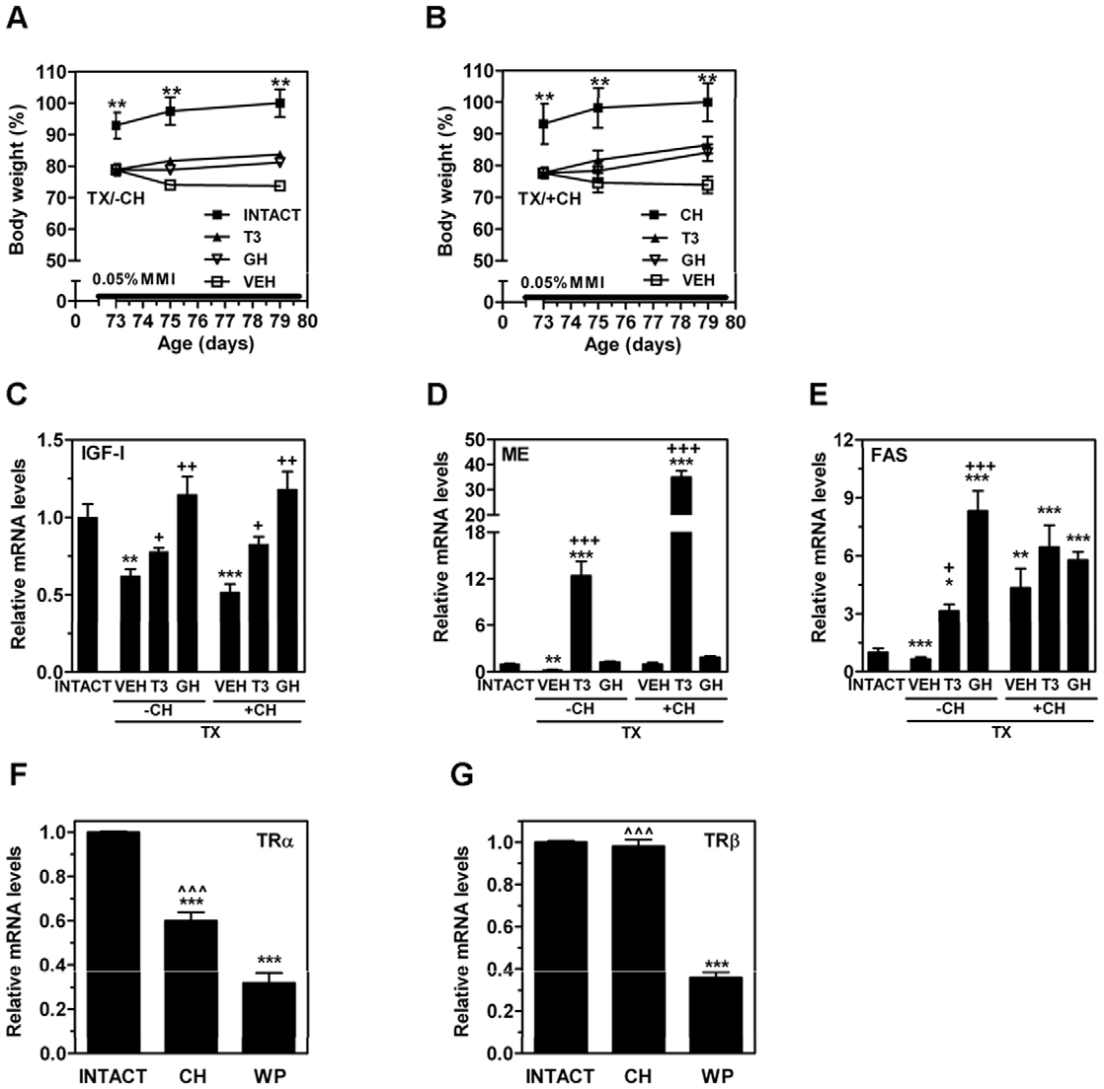
Effects of hormonal replacement on body weight and hepatic mRNA expression levels of IGF-I, ME, and FAS in adult hypothyroid rats without or with transient exposure to neonatal hypothyroidism. Four groups were studied: 1) age-matched rats (INTACT); 2) adult rats with neonatal hypothyroidism (CH); 3) hypothyroid adult rats without CH (TX/−CH); and 4) hypothyroid adult rats with CH (TX/+CH). During the last week of life, TX/−CH and TX/+CH groups were treated with either T3 or GH daily. Control animals were injected with saline (VEH). Body weight (A and B) as well as hepatic mRNA levels of IGF-I (C), ME (D) and FAS (E) were measured. The hepatic mRNA levels of TRα (F) and TRβ (G) were also measured by qPCR in CH, age-matched INTACT or weight-paired (WP) control groups. The mean mRNA expression level of each gene in the INTACT group is defined as 1, with all other expression values reported relative to this level. Results represent mean ± SD from at least six individual rats *, *P*<0.05; **, *P*<0.01; ***, *P*<0.001 for comparison with INTACT group (panel A) and for comparison with CH group (panel B); +, *P*<0.05; ++, *P*<0.01; +++, *P*<0.001 for comparison with vehicle-treated TX group. **^̂̂**, *P*<0.001 for comparison with WP group.

Although CH rats were euthyroid on PND80 (i.e., the time when the comparative gene expression was performed), they showed changes in liver gene expression in comparison with age-matched INTACT rats (see above) that involved previously characterized T3 (e.g., ME) or GH (e.g.SOCS2, CIS, IGF-I, CYP2C11, INSIG-1, and IGFBP-3) regulated genes. Thus, we hypothesized that the different transcriptional profile in the CH group could be explained by altered tissue responsiveness to T3 and/or GH, two hormones that are drastically reduced by hypothyroidism [6], [7], [8]. To test this hypothesis, we developed a second burst of hypothyroidism (TX) in adulthood, which was followed by hormone replacement as described in “Materials and Methods”. As expected, in vehicle or GH-treated TX rats, the serum levels of T3 were significantly reduced whereas the levels of T3-replaced animals did not differ from the INTACT control rats (data not shown). We showed a decrease in the total body weights of TX rats (*P*<0.001) and little subsequent weight gain in the TX/−CH (Fig. 7A) and TX/+CH rats (Fig. 7B). Independent of CH status, we showed the following: 1) the development of TX increased the circulating cholesterol levels and decreased the triglyceride serum levels (Table 1), which were mainly due to an increase of LDL and HDL cholesterol and a decrease of VLDL (data not shown), respectively, while the T3 hormone replacement restored the circulating cholesterol; 2) T3 and GH treatments increased the body weight gain in TX rats but it was unable to normalize it completely (Fig. 7A,B); and 3) T3 and GH treatments increased the hepatic level of IGF-I mRNA and, unlike T3, GH was capable of fully restoring the level to normal (Fig. 7C). However, the development of TX in the CH group (i.e., TX/+CH) resulted in a greater than 3-fold reduction of circulating FFAs, which was not observed in the TX/−CH group, and the T3 replacement restored it (Table 1). Next, we evaluated the effects of hormone replacement on the hepatic lipid content (Table 2). Independent of the CH status, T3 treatment restored hepatic cholesterol levels. However, in comparison with INTACT, GH increased the hepatic cholesteryl esters in the TH/−CH but not in the TH/+CH group. Additionally, to evaluate whether an altered response to the T3 treatment was associated with an altered transcriptional response, we measured the changes in gene expression of T3-regulated genes such as ME and FAS. The effect of GH was also measured. As expected, the development of TX in TX/−CH rats significantly reduced the mRNA levels of ME (Fig. 7D) and FAS (Fig. 7E). However, the development of TX in the TX/+CH group did not decrease ME (Fig. 7D) and, surprisingly, increased the mRNA levels of FAS up to 5-fold (Fig. 7E). Furthermore, T3 replacement in the TX/−CH rats increased the mRNA expression levels of ME and FAS by 12- and 3-fold, respectively. However, T3 replacement in the TX/+CH rats increased the expression of ME up to 30-fold which suggested an altered tissue sensitivity to T3 replacement. Taken together, these findings suggest that the tissue responsiveness to TH was altered in rats previously exposed to CH. At this point, we can only speculate about the molecular mechanisms that could support our hypothesis. However, because altered hepatic levels of TR might support a different metabolic response to TH in the liver [12], [14], [59], we made an exploratory analysis of the expression of TRβ/TRα mRNA in the CH rats. The hepatic mRNA level of TRα (Fig. 7F) was significantly reduced in the CH group whereas the TRβ mRNA (Fig. 7G) remained unaltered. However, the mRNA expression levels of TRα and TRβ were downregulated in the WP group, which suggests that TRα, unlike TRβ was influenced by CH.

**Table 1:** Serum lipids from PND80 male rats at baseline (INTACT), without (−CH) or with (+CH) transient neonatal exposure to MMI, during thyroid hormone deprivation (vehicle) and hormonal replacement.

	Cholesterol (mM)	Triglycerides (mM)	FFA (mM)
INTACT	1.38±0.13	1.59±0.65	0.69±0.17
TX/−CH			
Vehicle	3.01±0.13***	0.62±0.17**	0.58±0.17
T3	1.69±0.16+++	0.67±0.20**	0.75±0.30
GH	2.77±0.39***	0.44±0.13***	0.28±0.04***; ++
TX/+CH			
Vehicle	3.15±0.31***	0.72±0.05**	0.24±0.07***
T3	1.52±0.18+++	0.61±0.17**	0.59±0.13+++
GH	2.70±0.57***	0.66±0.20**	0.25±0.06***

**INTACT and CH animals were exposed to MMI at PND60 as described in Materials and Methods. From day 73, animals were injected daily with vehicle, T3 or GH for 7 d. The animals were sacrificed on PND80 and serum lipids were measured. Results are expressed as mean ± SD (n = 6). Statistical comparison was performed for treated animals using INTACT animals or vehicle as controls.**

** **,** ***P*** **<0.01;**

*** **,** ***P*** **<0.001 for comparison with INTACT rats;**

++ **,** ***P*** **<0.01;**

+++ **,** ***P*** **<0.001 for comparison with vehicle.**

**Table 2:** Hepatic lipids in liver from PND80 male rats at baseline (INTACT), without (−CH) or with (+CH) transient neonatal exposure to MMI, during thyroid hormone deprivation (vehicle) and hormonal replacement.

	Free cholesterol (mg/g of tissue)	Triglycerides (mg/g of tissue)	Cholesteryl esters (mg/g of tissue)	Fatty acids (mg/g of tissue)	Phospholipids (mg/g of tissue)
INTACT	2.4±0.2	3.9±0.6	0.30±0.02	0.18±0.03	57.8±4.1
TX/−CH					
Vehicle	1.9±0.1**	2.1±0.2***	0.37±0.04*	0.15±0.01*	51.6±3.4*
T3	2.3±0.3++	1.5±0.2***;+	0.33±0.05	0.14±0.01**	58.2±5.9
GH	2.1±0.2**	1.4±0.1***;++	0.54±0.15 ***;++	0.12±0.01***;+	50.7±4.9*
TX/+CH					
Vehicle	1.9±0.2**	2.1±0.3***	0.21±0.02**	0.13±0.01**	48.9±4.8*
T3	2.0±0.3*	1.7±0.4***	0.34±0.09++	0.13±0.01**	54.6±5.5
GH	1.7±0.2***	1.6±0.3***	0.31±0.07++	0.12±0.01**	43.8±2.3***;+

**INTACT and CH animals were exposed to MMI at PND60 as described in Materials and Methods. From day 73, animals were injected daily with vehicle, T3 or GH for 7 d. The animals were sacrificed on PND80 and hepatic lipids were measured. Results are expressed as mean ± SD (n = 6). Statistical comparison was performed for treated animals using INTACT animals or vehicle as controls.**

* **,** ***P*** **<0.05;**

** **,** ***P*** **<0.01;**

*** **,** ***P*** **<0.001 for comparison with INTACT rats;**

+ **,** ***P*** **<0.05;**

++ **,** ***P*** **<0.01 for comparison with vehicle.**

## Discussion

The THs are essential for development, growth, and metabolism [1], [2], [3]. The present study shows that transient neonatal hypothyroidism in male rats gave rise to endocrine alterations that not only affected postnatal growth but also influenced hepatic physiology and responsiveness to THs replacement in adulthood.

Growth-inhibiting conditions exist during development in association with malnutrition, glucocorticoid excess, systemic diseases, GH-IGF-I deficiency, or hypothyroidism [44], [60], [61]. In this work, several biomarkers of neonatal hypothyroidism (i.e., decreased circulating THs and hepatic mRNA expression levels of ME and Spot14) and high expression of IGFBP-2 were associated with decreased circulating IGF-I and a delayed somatic growth rate on PND30. Furthermore, when the growth-inhibiting condition (i.e., MMI) was removed, somatic growth rate (weight and tail lenght gain) and food efficiency increased in CH rats, which is a phenomenon known as catch-up growth [44], [60]. By PND80, however, this effect had subsided, and somatic growth rate (see body weight gain and tail length gain in Supplementary File S3) in CH animals was similar to age-matched INTACT controls. Alternatively, higher food efficiency remained in euthyroid CH group on PND80 along with significance differences in total body weight and size in comparison with INTACT group, which suggests, in agreement with previously reported data [62], a higher but less efficient rate of metabolism (i.e., a reduced ability to transform calories consumed into total body weight and size) in rats previously exposed to CH. The CH rats on PND80 also showed increased mRNA levels of several GH target genes (i.e., IGF-I, SOCS-2, CIS, CYP2C11, CYP2C13) suggesting that the increased hepatic GH activity observed in these animals was possibly associated with catch-up growth. In contrast, other well-known GH target genes in female rats such as CYP2C7 and CD36 were downregulated in CH group. This apparent paradox could be explained by sexually dimorphic pattern of gene expression in rat liver [63], [64]. The downregulation of female-predominant genes (e.g., CYP2C7 and CD36) concomitant with the induction of male-predominant genes (e.g., CYP2C11 and CYP2C13) suggests that a male pattern of gene expression was enhanced in CH rat liver.

In the current study, we show that transient CH is associated with changes in SOCS-2 and CIS expression, which are key negative regulators of GH-dependent somatic growth *in vivo* [51], [65]. GH resistance can be shown in rat models of sepsis and uremia and in small rats for gestational age (SGA) without catch-up growth. This was associated with an increased expression of SOCS-2 and CIS and impaired JAK/STAT signaling [66], [67], [68]. In our model, however, catch-up growth was associated with the overexpression of SOCS-2 and CIS in adult CH rats. Whether the overexpression of SOCS and CIS is associated with delayed growth development and catch-up growth in CH rats requires further research.

Growth-inhibiting conditions during fetal-neonatal period of life may influence lipid metabolism in adulthood [69], [70], [71], [72]. Human and rats, who do show catch-up of somatic growth and increased feed efficiency after withdrawal of growth-inhibiting condition (e.g., SGA or caloric restriction), have higher risk of fat in the liver and increased adiposity in adulthood [69], [70], [71], [72]. Now, we show that somatic growth inhibition by neonatal hypothyroidism influences hepatic lipid metabolism in adulthood. CH rats showed a concomitant upregulation of PPARα and CPT1, a gene related to fatty acid catabolism. Furthermore, adult CH rats showed a downregulation of CD36, which is involved in fatty acid uptake and a well-known PPARα target gene, along with the reduced transcription of genes involved in cholesterol uptake (LDLR), cellular sterol efflux (ABCA), triglyceride assembly (MTTP), bile acid synthesis (CYP8B1, CYP7A1 and CYP27A1), and lipogenesis (SREBP1c) [73] . These data indicate that CH significantly influenced lipid metabolism in adulthood and, most likely, contributed to the diminished hepatic levels of triglycerides, cholesteryl esters, and FFA. Conversely, because expression levels of several lipid genes in CH adult group were altered in similar direction to that detected in WP group, our data could be explained, in part, as a consequence of delayed growth [33], [34]. Furthermore, a reduced content of hepatic lipids in CH group could be caused by prolonged catch-up growth which might cause an increased lipid catabolism in growing animals (i.e., CH group) in comparison with those that have completed their body growth (i.e., INTACT). To determine whether some of these changes caused by CH are life-long adaptations, similar analysis would need to be performed in older animals [33], [34]. Furthermore, the reduced content of hepatic lipids in CH group could be caused by prolonged catch-up growth which might cause increased lipid catabolism in growing animals (i.e., CH group) in comparison with those that have completed their body growth (i.e., INTACT). However, several reports have suggested that catch-up growth is likely associated with increased level of hepatic lipids and adiposity [72], which would not be in agreement with this explanation. Additionally, despite all of the changes observed in liver, the levels of circulating lipids (triglycerides and cholesterol) and lipoproteins (data not shown) were similar to those in the INTACT aged-matched littermates, which suggest that CH rats were able to maintain lipid homeostasis and support the increased energy demands imposed by an accelerated growth rate. This is apparently achieved by redistributing lipids from the liver towards peripheral tissues rather than through active hepatic lipogenesis, which is an energy-consuming process that would compete with peripheral energy needs.

In this study, we show that transient neonatal hypothyroidism influences transcriptional program in adult liver. Despite being euthyroid, adult CH animal showed a modified transcriptional profile in liver in comparison with age-matched INTACT rats, which might be explained by altered tissue responsiveness to T3 and/or GH, two hormones that are drastically reduced by hypothyroidism [6], [7], [8]. However, independent of CH status, several of the responses to hypothyroidism and hormone replacement were similar. As expected, development of TX increased circulating cholesterol levels and decreased the serum triglyceride levels, while T3 hormone replacement restored circulating cholesterol level. Additionally, T3 and GH treatments increased the body weight gain and hepatic levels of IGF-I mRNA. At first glance, these results suggested that the CH rats, after suffering a biological insult (i.e., a second burst of hypothyroidism in adulthood), showed a biological response similar to age-matched INTACT rats. However, several of the responses to TX or hormone replacement suggested an altered lipid metabolism in the CH rats. First, a significant reduction of circulating FFAs by TX in TX/+CH group but not in TX/−CH group, an effect that was restored by T3 replacement. Second, GH-increased hepatic cholesterol esterification occurred in the TH/−CH animals but not in the TH/+CH animals. Third, GH treatment reduced serum VLDL fraction in the TX/−CH but not in the TX/+CH rats (data not shown). We did not observe major alterations in hepatic reactivity to GH in terms of lipid changes which suggests that the capacity of GH treatment to reduce serum triglyceride levels in TX/−CH rats, but not in TX/+CH rats, is most likely due to altered GH activity in extrahepatic tissues, such as fat and muscle [3]. Finally, hepatic concentrations of lipids in T3-treated TX/+CH rats did not differ significantly from TX/−CH group, which suggested that the homeostatic capacity of CH tissue in response to T3 was not dramatically affected. However, this is in contrast to the enhanced ME expression in the TX/+CH group in response to the T3 treatment. Increased ME mRNA expression did not seem to be a general response to T3 replacement because SREBP1c and 2 showed a less pronounced change (data not shown). ME is directly regulated by the binding of TR to a TRE in the promoter of the ME gene [74]. Our measurement of mRNA levels for TR receptors in liver showed unaltered expression of the major isoform TRβ and reduced levels of TRα, making it unlikely that changes in ME expression can be attributed to altered TR content. Noticeably, the ME regulatory region also contains binding sites for PBX1 and 2 [75], CEBPα [76], and an E-box [77] that can modulate the response to T3. At this point, we cannot exclude the possibility that CH effects on these transcription factors and other nuclear co-regulators influence ME expression but additional experiments are needed to test this hypothesis. Likewise, a clearer mechanistic explanation for the metabolic changes observed in the CH rats would require, among others measures, the analysis of fat and muscle metabolism.

Androgens may influence the hepatic response to CH. It is well known that neonatal hypothyroidism results in increased circulating levels of testosterone in male rats, which is secondary to increased testis size [29]. We also observed two-fold higher serum testosterone levels in the CH rats compared to the age-matched adult INTACT rats (data not shown). Although the liver is not considered to be a primary target of testosterone action, androgens maintain specific male pattern of pituitary GH secretion and actions on liver [78], and it has been shown that specific deletion of androgen receptor (AR) in liver of male animals causes hepatic insulin resistance with decreased fatty acid β-oxidation and steatosis [79], which implicates the hepatic AR as a positive factor in maintaining physiological control of glucose and lipid homeostasis. Alternatively, in prostate, a well-known target tissue of androgen action, testosterone has lipogenic effects, such as inducing the expression of FAS [80]. Therefore, we cannot exclude the possibility that some of the transcriptional effects detected in the liver of the CH rats (e.g., increased CYP2C11, CYP2C13, and PPARα) were secondary to increased levels of circulating testosterone.

In summary, our findings support the hypothesis that TH deprivation during neonatal period of life causes long-lasting influence on the liver transcriptome and provokes an altered responsiveness to biological insult in adulthood. Several findings that cannot be explained by the lower body weight in CH rats, compared to WP, include genes regulated by GH (e.g., IGF-I, CIS, CYP2C11, and CYP2C13) and genes involved in hepatic lipid metabolism (e.g., PPARα, CPT1, ME, ACC1, FAS, CYP4F1, AOX, L-FABP, LXR, HMGCoA-S, and HMGCoA-R). Being clinically relevant, the changes observed in the transcriptional responses to T3 highlight the possibility that CH influences tissue reactivity to thyromimetic drugs in adulthood [81]. Interestingly, thyroid-disrupting compounds, which can cause neonatal hypothyroidism, include a wide range of chemicals from naturally occurring compounds, pharmaceuticals, and a number of xenobiotics [82]. The long-lasting influence of growth-inhibiting conditions on hepatic metabolism is intriguing and warrants further study to explore whether the alterations observed in this study cause metabolic disruptions or chronic diseases.

## Supporting Information

File S1 **Schematic diagram of rat model used to study the effects of congenital hypothyroidism on adult rat liver.** Congenital-neonatal hypothyroid male rats (CH) were produced by 0.02%-MMI administration in the drinking water of pregnant rats (GD12) until weaning at PND30. For generation of adult hypothyroid rats (TX), 0.05% MMI was added to the drinking water for 3 weeks starting at PND58. Four groups were studied: 1) euthyroid age-matched rats (INTACT); 2) CH; 3) TX rats without CH (TX/−CH); and 4) TX rats with CH (TX/+CH). During the last week of life, TX/−CH and TX/+CH groups were treated with either T3 or GH daily for 7 days as described in Materials and Methods. Control animals were injected with saline. Each group included six individual animals.
(TIF)

File S2 **Gene names and primer sequences (5′- 3′) used for real-time PCR.**
(TIF)

File S3 **Effects of neonatal hypothyroidism on body growth development.** Body weight (A) and tail length (B) were measured at 7-d intervals. On PND30, the hepatic mRNA levels of IGFBP2 (C) were measured by qPCR in rats exposed to neonatal hypothyroidism (CH), age-matched (INTACT) or weight-paired (WP30) control groups. Body weight gain (D), tail growth gain (E) and food efficiency (F) were measured at 7-d intervals as described in Material and Methods. Results are expressed as mean ± SD from six individual animals in each group. **, *P*<0.01, ***, *P*<0.001 for comparison with INTACT group. **+++**, *P*<0.001 for comparison with WP group.
(TIF)
