# Supplementary figures and images for: Influence of Neonatal Hypothyroidism on Hepatic Gene Expression and Lipid Metabolism in Adulthood
Source: PLoS One. 2012 May 16;7(5):e37386. doi: 10.1371/journal.pone.0037386 (PMC3354003; doi:10.1371/journal.pone.0037386)

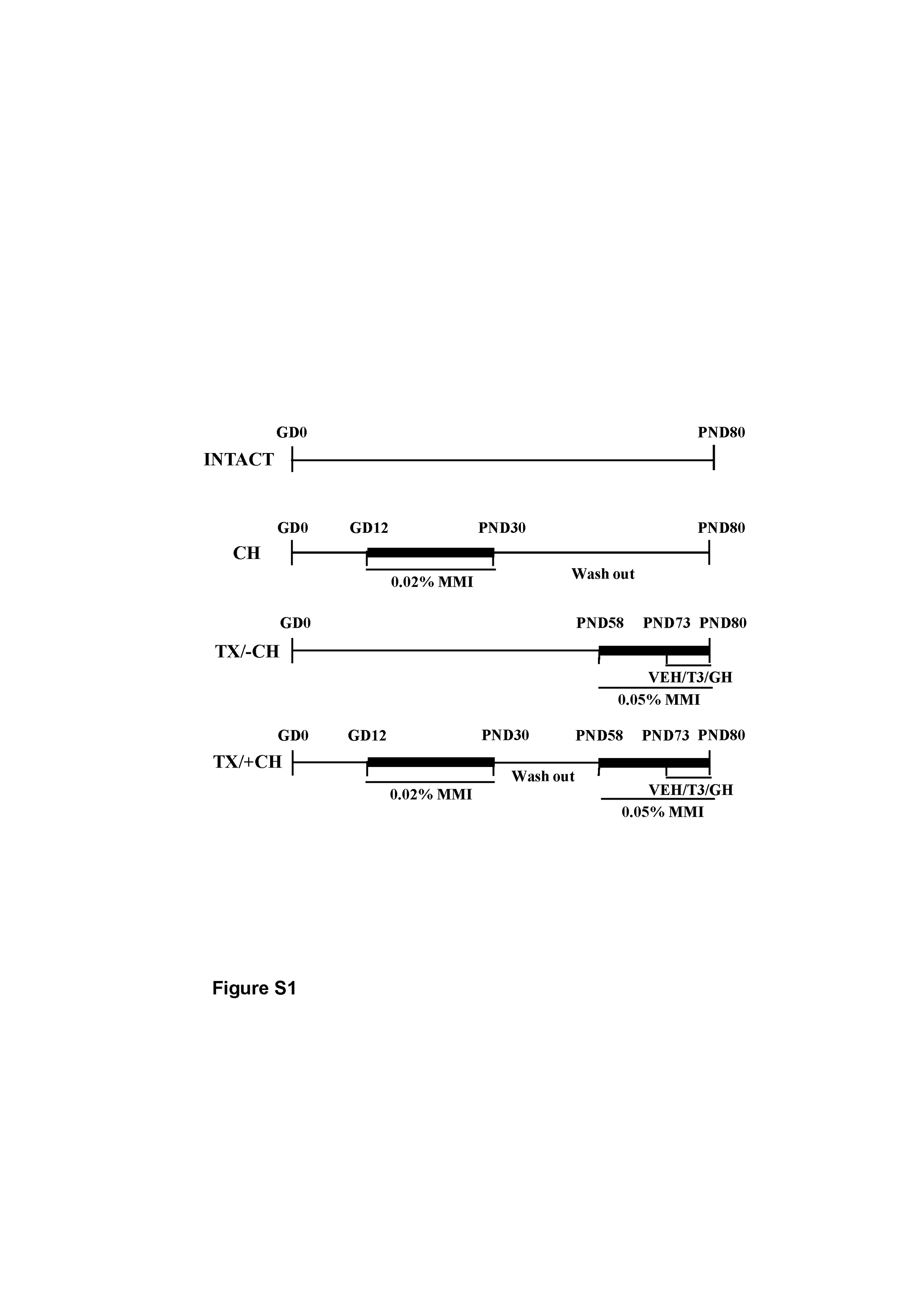

Supplement: File S1 — Schematic diagram of rat model used to study the effects of congenital hypothyroidism on adult rat liver. Congenital-neonatal hypothyroid male rats (CH) were produced by 0.02%-MMI administration in the drinking water of pregnant rats (GD12) until weaning at PND30. For generation of adult hypothyroid rats (TX), 0.05% MMI was added to the drinking water for 3 weeks starting at PND58. Four groups were studied: 1) euthyroid age-matched rats (INTACT); 2) CH; 3) TX rats without CH (TX/−CH); and 4) TX rats with CH (TX/+CH). During the last week of life, TX/−CH and TX/+CH groups were treated with either T3 or GH daily for 7 days as described in Materials and Methods. Control animals were injected with saline. Each group included six individual animals. (TIF) [file pone.0037386.s001.tif]

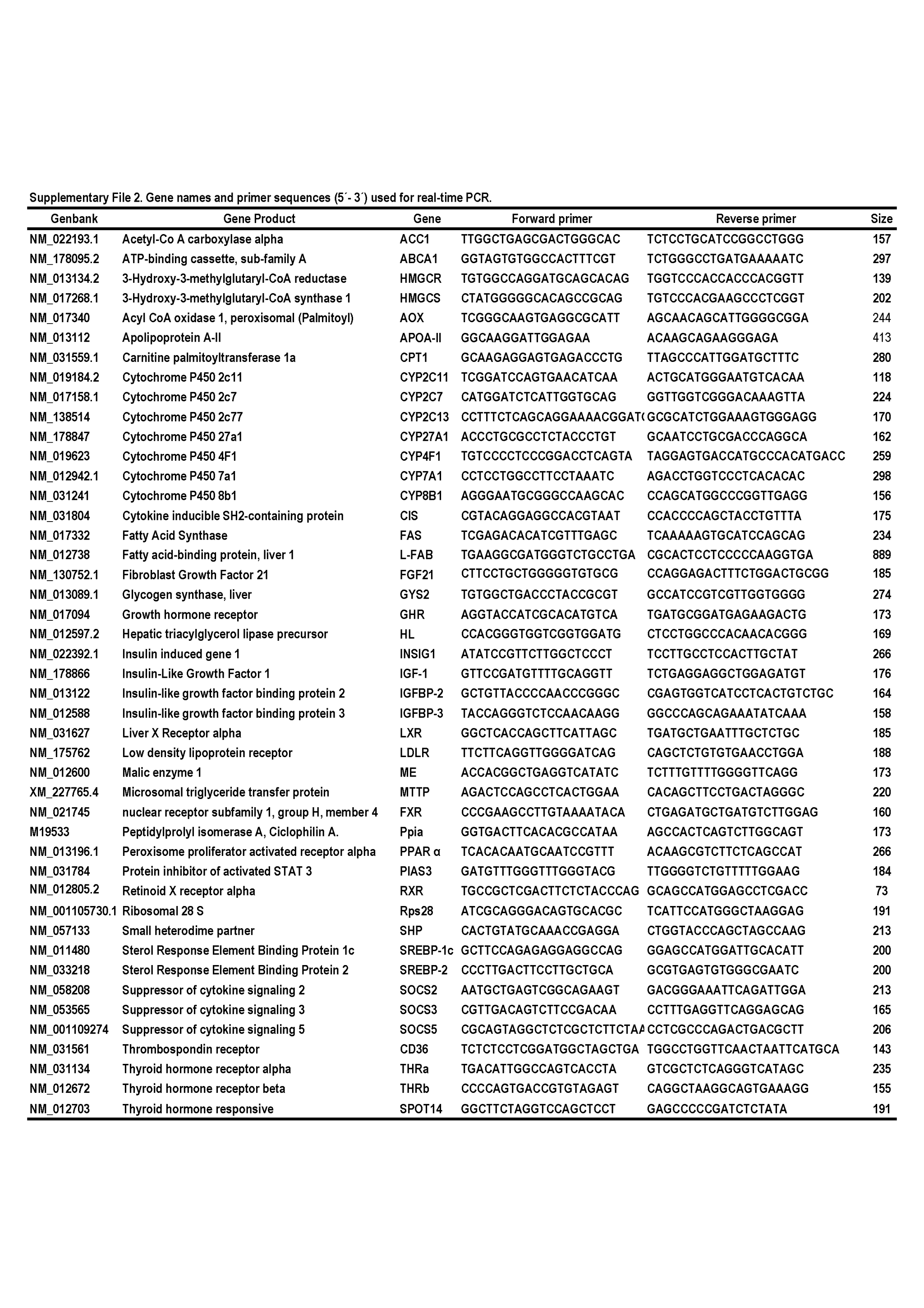

Supplement: File S2 — Gene names and primer sequences (5′- 3′) used for real-time PCR. (TIF) [file pone.0037386.s002.tif]

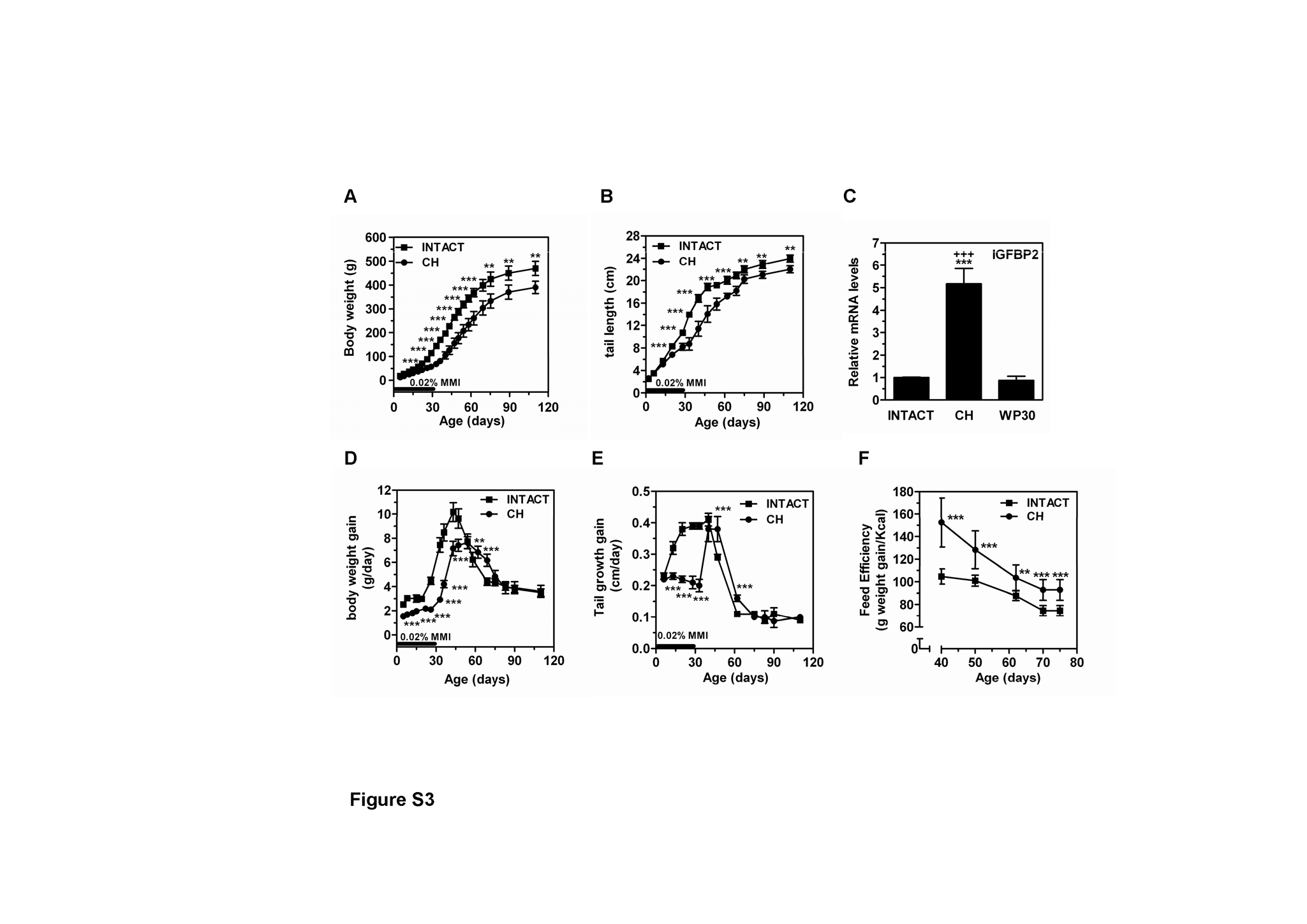

Supplement: File S3 — Effects of neonatal hypothyroidism on body growth development. Body weight (A) and tail length (B) were measured at 7-d intervals. On PND30, the hepatic mRNA levels of IGFBP2 (C) were measured by qPCR in rats exposed to neonatal hypothyroidism (CH), age-matched (INTACT) or weight-paired (WP30) control groups. Body weight gain (D), tail growth gain (E) and food efficiency (F) were measured at 7-d intervals as described in Material and Methods. Results are expressed as mean ± SD from six individual animals in each group. **, P<0.01, ***, P<0.001 for comparison with INTACT group. +++, P<0.001 for comparison with WP group. (TIF) [file pone.0037386.s003.tif]
